# Prevalence and risk factors of human *Balantidium coli* infection and its association with haematological and biochemical parameters in Ga West Municipality, Ghana

**DOI:** 10.1186/s12879-021-06731-2

**Published:** 2021-10-09

**Authors:** Enoch Aninagyei, Salifu Nanga, Desmond Omane Acheampong, Rita Mensah, Mercy Nelly Boadu, Henrietta Terko Kwansa-Bentum, Clement Okraku Tettey

**Affiliations:** 1 School of Basic and Biomedical Sciences, Department of Biomedical Sciences, University of Health and Allied Sciences, PMB 31 Ho, Volta Region Ghana; 2 School of Basic and Biomedical Sciences, Department of Basic Sciences, University of Health and Allied Sciences, PMB 31 Ho, Volta Region Ghana; 3 Department of Biomedical Sciences, School of Allied Health Sciences, University of Cape Coast, Cape Coast, Ghana; 4 Ghana Health Service, Ga North Municipal Hospital, Ofankor, Accra Ghana; 5 Department of Nursing and Midwifery, Faculty of Health and Allied Sciences, Pentecost University, Accra, Ghana

**Keywords:** *Balantidium coli*, Balantidiosis, Eosin-saline wet preparation, Formol-ether concentration technique, Ga West Municipality, Ghana

## Abstract

**Background:**

In Ghana, *Balantidium coli* (*B. coli*) has been identified in vegetables and in pigs, although there is a paucity of data regarding human balantidiosis. This study sought to assess human *B. coli* infection in Ghana, factors associated with the infection as well as its association with haematological and biochemical parameters.

**Methods:**

Two pig rearing communities in the Ga West Municipality, Ghana, were involved in this study. Stool and blood samples were collected from pig farmers and their exposed household members as well as relevant information on potential associated factors. Eosin-saline wet preparation was done on the same day of stool samples were collected while formol ether concentration technique was performed later. Haematological, biochemical parameters and serum electrolytes were determined using Celltac MEK-6500 K, PKL-125 biochemical analyser, and FT-320 electrolyte analyser, respectively.

**Results:**

The overall prevalence of balantidiosis was 10.4 %, significantly higher among farmers (21.7 %) than in exposed household members (5.8 %) (*x*^2^ = 17.8, p = 0.000025). Of the 43 infected individuals, 20.9 % were co-infected with either *Entamoeba histolytica, Giardia lamblia*, or *Schistosoma mansoni.* In *B. coli* infection, mild to moderate anaemia together with a reduction in levels of platelet, albumin and, sodium, chloride, and bicarbonate ions were observed. However, white blood cells were significantly elevated in infected states. Poor farming practices such as free-range systems, improper disposal of pig faeces, lack of use of protective farming clothing, and unavailability of dedicated farming clothing were found to be associated with *B. coli* infection status. Finally, frequent diarrhea (OR = 12.30, p = 0.006) with occult blood (OR = 25.94, p < 0.0001) were found to be predictors of *B. coli* infection.

**Conclusions:**

Human balantidiosis is endemic in Ga West Municipality, Ghana. Individuals living closed to pig rearing communities presenting with frequent diarrhea with occult blood in stool should be screened and treated for balantidiosis to mitigate the clinical consequences of the infection.

## Background

Data on human balantidiosis, caused by the only known human ciliate, *Balantidium coli* (*B. coli*), is lacking in Ghana. However, previous studies have identified *B. coli* in commonly consumed vegetables in Ghana. Water supply containing pig faeces used for irrigation being the main source of contamination [1]. In the Upper East region of Ghana, almost 20 % of pigs were found to harbour *B. coli* [2]. The presence of parasites in vegetables and in pigs could serve as a source of human infections.

*B. coli* is known to be transmitted to humans by domesticated pigs [3], where transmission occurs by ingestion of cysts through direct or indirect contact and through other means such as contaminated food and water by human and pig faeces [4]. After encystment in the intestines, the motile trophozoites attack the intestinal epithelium, creating ulcers. *B. coli* is able to cause ulcers by secreting an enzyme called hyaluronidase. This enzyme is capable of degrading intestinal tissues and facilitates penetration of the mucosa. *B. coli* causes bloody diarrhea which is similar to that of amoebic dysentery [5]. Some cardinal symptoms of balantidiosis in humans include passing of loose stools, anorexia, fever, and mild abdominal pain [6]. Currently, Centre for Disease Control has recommended the use of tetracycline, metronidazole, and iodoquinol as the treatment option for human balantidiosis [7].

In Ghana, pigs are reared in many parts of the country for nutritional and economic purposes. Due to the lack of advanced technology in rearing pigs in Ghana and other parts of Africa, most farmers employ the services of household members and other community members to support pig production. Most piggeries are done in sheds and stalls, whereas others are free animals with some co-habiting with humans. Feeding is done manually and droppings were used as manure or indiscriminately discarded [8]. These practices are consistent with factors associated with increased transmission of *B. coli* [3]. However, there is limited data regarding balantidiosis in Ghanaians. Even though the global prevalence of balantidiosis is very low (0.02–1 %) [9], the clinical correlates of this neglected tropical/subtropical infection must be established. In furtherance to the foregoing, the objective of this study was to assess human *B. coli* infection and associated clinical factors in Ghana, a sub-Saharan country located in West Africa.

## Methods

### Study design, study sites, and period of sampling

This purposive cross-sectional study was conducted between February–May, 2020. This study was carried out in two pig rearing communities, Opah and Onyansana, two villages which are farming communities in the Ga West Municipality in the Greater Accra region of Ghana. A recent parasitological survey reported 1.6 % prevalence of balantidiosis in these two communities [10].

### Selection of study participants

Individuals engaged in pig farming as well as their household members were recruited in this study. In each study area, with the help of community-based surveillance volunteers (CBSV), all pig farmers were identified. The pig farmers then led the research team to their houses to interact with their exposed household members. An exposed household member was defined as anyone who did not actively take part in pig farming but frequently entered the pigsty for one reason or the other as well as those that came into contact with pig droppings and pig faeces soiled farming accoutrement.

### Sample size estimation

Using the Cochrane’s formula, n = z^2^p(1-p)/d^2^, where n is the sample size, *z* is the confidence level at 95 % (standard value of 1.96), d is the margin of error at 5 % (standard value of 0.05), the sample size was 384, assuming the prevalence of balantidiosis in pig farming communities was 50 % [11].

### Inclusion and exclusion criteria

Individuals included in this study were pig farmers and exposed household members. Individuals negative for *B. coli* were randomly selected as a noninfected comparative group in a ratio of two noninfected individuals to one infected individual. Household members that did not support farming activities, declined blood sample collection, or did not meet the definition above were not included.

### Stool and blood sample collection and relevant information collection

For both pig farmers and their household members, a minimum of 2 g of early morning stool sample was collected as well as whole blood samples in both K_2_-EDTA (~ 3 mL) and clot activator tubes (~ 4 mL). Stool samples were kept at ambient temperature while whole blood samples were kept in coolers containing ice packs prior to arrival in the Ga North Municipal Hospital Laboratory located in Ofankor-Accra. Both structured questionnaires and personal observations were used to elicit responses on the type of housing for the pigs, disposal system for pig droppings, disinfection protocol after leaving the pigsty, use of protective equipment (boots, gloves, face shields and goggles), availability of dedicated farming clothing, and history of deworming the pigs. Enrolees were also asked whether there were restricted entry protocols in place, whether farmers and their household members got soiled with pig droppings when cleaning the pigsty. Information regarding the number of times the farmers enter the pigsty and the frequency of washing farming clothing were also collected.

### Physical examination and detection of occult blood in stool samples

Stool samples were physically examined for consistency based on the amount of water present in the stool. Formed specimens were stool samples with solid appearance (with little amount of water), semi-formed specimens were solid samples with adequate amount of water, while the loose specimens did not have visible water but able to spread to take the shape of its container. Mucoid specimens contained visible mucus while blood was observed in bloody specimens. Occult blood was determined by using the blood determinant portion of Uritest 10E Urine reagent strip (URIT Medical Electronics Co. Ltd, Guangxi, China) following the manufacturer’s procedure.

### Processing of stool samples and analysis of blood specimens

On the same day stool samples were collected, direct wet preparation using eosin saline was performed. The rest of the stool sample was fixed in 10 % buffered formalin. Additionally, haematological parameters were determined using Celltac MEK-6500 K automated analyser (Nihon Kohden, Japan). Serum and plasma samples were stored at − 30 °C until analysis. Serum electrolytes (sodium ions, potassium ions, and chloride ions) were measured by FT-320 electrolyte analyzer (Fortune Co. Ltd, China), while plasma urea, creatinine, ALP, total protein, albumin, AST, ALT, indirect bilirubin, and direct bilirubin were measured by PKL-125 (Paramedical Co. Ltd, Italia) fully automated chemistry analyser. For all the biochemical analysis, Medsource reagents (Ozone Biomedicals Pvt Ltd, Haryana, India) were used. Serum bicarbonate ions were measured spectrophotometrically based on the principle that bicarbonate ions react with phosphoenolpyruvate catalysed by phosphoenolpyruvate carboxylase to form oxaloacetate and phosphate. The oxaloacetate was then converted to malate by the action of malate dehydrogenase and reduced nicotinamide adenine dinucleotide (NADH). The decrease in absorbance at 415 nm resulting from the oxidation of NADH was proportional to the amount of bicarbonate in the sample.

### Morphologic characterization of *B. coli*

#### Detection of *B. coli* using eosin-saline mixture

Stool samples were emulsified into a uniform mixture with normal saline using a wooden spatula. A drop of the preparation was placed on a microscope slide and emulsified with saline and eosin solution (10 % v/v). The emulsified stool sample was examined using a bright field microscope (objectives ×10 and ×40). Viable parasites were identified by their characteristic shape and motility, while *B. coli* cyst was identified by its spherical or slightly ovoid shape.

#### Detection of *B. coli* cysts using the formol ether concentration (FEC) method

Stool samples were vortexed into a homogenous mixture; then, 1 mL of the homogenate was sieved using non-absorbent gauze. The sieved homogenate was centrifuged at 3,000 rpm after adding 7.0 mL of formol saline reagent and 3 mL of diethyl ether (Honeywell, USA: bp: 34.6 °C, mp: − 116.3 °C, mm: 74.12 g/mol). Supernatant was discarded and the pellet mixed with iodine solution. The pellet was examined with a bright field microscope, using objectives ×10 and ×40. Parasite cysts were identified by their characteristic spherical or slightly ovoid shape.

### Data analysis

Prevalence of *B. coli* was expressed as the ratio of the number of infected individuals in a given category and the total number of individuals examined in that category. Fisher’s exact test was used to compare *B. coli* infection status between stool parameters (stool consistency and stool occult blood) and history of diarrhea. One-way ANOVA was used to compare haematological and biochemical parameters between *B. coli* mono-infected, *B. coli* co-infected, and noninfected individuals. In case of significant difference, Tukey post hoc multiple analysis was performed to determine the specific groups significantly different or similar. Univariate logistic regression analysis was used to determine predictors of *B. coli* infection. Data were analysed by SPSS Version 24 (Chicago, IL, USA). P-values less than 5 % were considered statistically significant.

## Results

### Characteristics of study participants

**Fig. 1:**
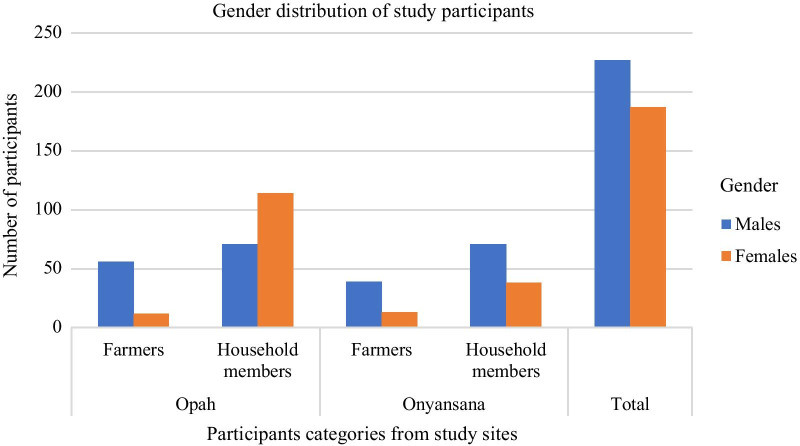
Gender distribution of the study participants

**Fig. 2:**
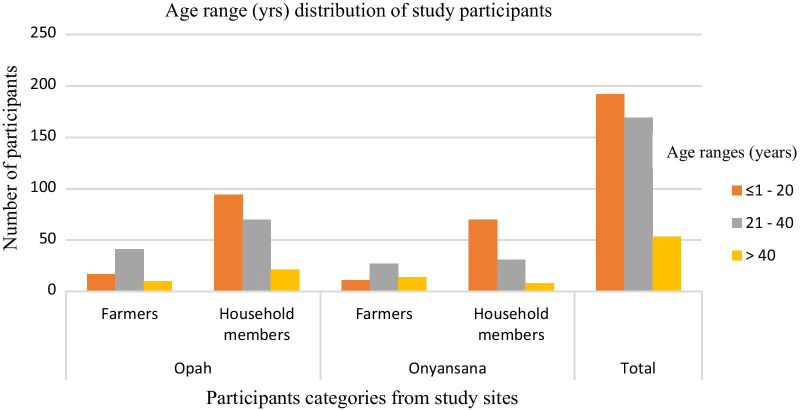
Age range distribution of the study participants

A total of 414 participants were recruited in this study, of which 120 (30.0 %) were pig farmers (age range: 16–71 years) while 294 (70 %) were exposed household members (age range: 6–73 years). At Opah, 253 (61.1 %) samples were collected from 15 affiliated farms, whereas 161 (38.9 %) samples were collected at Onyansana from 11 affiliated farms. In the two study sites, male farmers were more frequent (54.8 %), although female participants were more enrolled in Opah (61.6 %) (Fig. 1). Participants aged < 21 years (46.4 %)) were marginally more than those aged 21–40 years (40.8 %), between the two study sites and between farmers and their exposed household members; participants aged > 40 years were least represented (Fig. 2).

### Prevalence of balantidiosis in the study sites

Table 1 presents the infection rates between farmers and exposed household members. The overall prevalence of *B. coli* infection in the study sites was 10.4 %, significantly higher among pig farmers (21.7 %) compared to exposed household members (5.8 %) (*x*^2^ = 17.8, df = 1, p < 0.001). Distribution of *B. coli* infection in Opah was similar to that in Onyansana (*x*^2^ = 0.34, df = 1, p = 0.556). Likewise, the infection rate was similar among males and females (*x*^2^ = 0.72, df = 1, p = 0.393). However, balantidiosis infection was higher among participants aged 21–40-year-old (*x*^2^ = 7.9, df = 2, p = 0.019). In addition, human balantidiosis was associated with educational level. Aside *B. coli* infection, other parasites were also identified, namely, *Entamoeba histolytica* (n = 23, 5.6 %), *Giardia lamblia* (n = 19, 4.6 %) and *Schistosoma mansoni* (n = 7, 1.7 %). However, *B. coli* co-existed with only nine of the other parasites (6 *E. histolytica* and 3 *G. lamblia*).

**Table 1:** Infection with *Balantidium coli* according to demographic characteristics

	*B. coli* infected Farmers only (n = 26)	*B. coli* infected household members only (n = 17)	Overall participants (n = 414)		
Demographic characteristics	Number examined (%)	Number examined (%)	Number examined (%)	Prevalence^a^(%)	Fisher’s Exact Test (p–value)
Study sites					0.34 (0.556)
Opah	13 (50.0 %)	10 (58.8 %)	253	5.5 %	
Onyansana	13 (50.0 %)	7 (41.2 %)	161	4.8 %	
Gender					0.72 (0.393)
Males	19 (73.1)	10 (58.8)	237	7.0	
Females	7 (26.9)	7 (41.2)	177	3.4	
Age range (years)					7.9 (0.019)
≤ 1–20	9 (34.6 %)	7 (41.2 %)	192	3.9 %	
21–40	12 (46.2)	9 (52.9 %)	169	5.1 %	
> 40	5 (19.2 %)	1 (5.9 %)	53	1.4 %	
Highest education level					10.9 (0.011)
None	14 (53.8)	4 (23.5)	65	4.3	
Primary	3 (11.5)	6 (35.3)	120	2.2	
Junior high	7 (26.9)	6 (35.3)	169	3.1	
Senior high	2 (7.7)	1 (5.9)	60	0.7	

### *B. coli* infection status according to biophysical properties of stool sample

Among the various stool sample consistencies, semi-formed samples (p = 0.037), loose samples (p < 0.001), and bloody stool samples (p = 0.03) were significantly associated with *B. coli* infection. Additionally, occult blood in stool was significantly higher among individuals infected with *B. coli* (*x*^2^ = 68.1, p < 0.05) and whose history of diarrhea was infrequent (*x*^2^ = 9.3, p = 0.002) (Table 2).

**Table 2:** *B. coli* infection status according to biophysical properties of stool samples and frequency of diarrhea

Parameter	Non–infected n (%)	Infected n (%)	Fisher’s Exact Test (p–value)
Consistency			
Formed specimens	141 (38.0)	8 (18.6)	3.3 (0.067)
Semi–formed specimens	168 (42.3)	9 (20.9)	4.3 (0.037)
Loose specimens	52 (14.0)	21 (48.8)	18.4 (< 0.001)
Bloody specimens	10 (2.7)	4 (9.3)	4.6 (0.03)
Mucoid and bloody specimens	0 (0)	1 (2.3)	–
Occult blood			
Present	51 (13.7)	43 (100)	68.1 (< 0.001)
Absent	320 (86.3)	0 (0)	–
Diarrhea in recent time*			
Frequent	69 (18.6)	15 (34.9)	3.8 (0.05)
Not frequent	109 (29.4)	28 (65.1)	9.3 (0.002)
Not applicable**	193 (52.0)	0 (0)	–

*Frequent diarrhea is at least one episode of diarrhea a week. Not frequent diarrhea is at most one episode of diarrhea every fortnight; **No history of diarrhea in recent times

### Association of *B. coli* infection status with demographic parameters, stool sample characteristics, and frequency of diarrhea

*B. coli* infection status was not associated with study sites (OR = 1.03, 95 % CI: 0.461–2.30, p = 0.943). Although the odds of being infected was higher in participants aged 21–40 years, the infection status was not significantly associated with age group. Additionally, being a male did not increase the risk of *B. coli* infection. Furthermore, participants with no formal education had higher odds of being infected, even though the infection status was not significantly associated with educational level. Similarly, stool consistency was not associated with infection status. However, frequent diarrhea (OR = 12.30, 95 % CI: 10.80–36.62, p = 0.006) with occult blood (OR = 25.94, 95 % CI: 11.9–56.4, < 0.0001) were found to be good predictors of *B. coli* infection in endemic communities (Table 3).

**Table 3:** Logistic regression analysis of factors associated with *B. coli* infection status

Study variables	OR	95 % CI	P–value
Study site			
Onyansana	1		
Opah	1.030	(0.45, 2.30)	0.943
Age group			
≤ − 20	1		
21–40	1.607	(0.63, 4.07)	0.317
> 40	1.290	(0.37, 4.51)	0.690
Gender			
Female	1		
Male	1.337	(0.62, 2.87)	0.455
Educational level			
Junior high	1		
None	1.476	(0.51, 4.24)	0.469
Primary	0.495	(0.16, 1.49)	0.212
Senior high	1.040	(0.29, 3.72)	0.952
Stool consistency			
Bloody	1		
Formed	0.700	(0.13, 3.89)	0.683
Loose	2.108	(0.37, 11.93)	0.399
Semi–formed	0.733	(0.15, 3.62)	0.703
Occult blood			
Absent	1		
Present	25.940	(11.93, 56.38)	< 0.001
Frequency of diarrhea			
Not frequent	1		
Frequent	12.30	(10.80, 36.62)	0.006
Not applicable	1.217	(0.48, 3.12)	0.681

### Animal husbandry practices in the farmed observed

Table 4 presents some animal husbandry practices identified among the pig farmers. Among the 26 pig farms, 65.4 % operated a caged system (pigsty), while 26.9  and 7.7 % were partially housed and not housed, respectively. Of the 24 farms where animals were either confined or partially confined, 54.2 % of the farmers indicated that the pig droppings were used as manure while 37.5 % of the farmers washed the pig faeces into drains; about 8 % of the farmers left faeces in the pigsty. Of the 26 infected pig farmers, 88.5 % did not disinfect themselves after leaving the pigsty while the majority of them did not use protective boots and gloves (73.0  and 88.5 %, respectively) while attending to the pigs. None of the infected farmers used a face mask as well as a goggle while attending the animals. Additionally, the farmers did not deworm the pigs (Table 4).

**Table 4:** Animal husbandry practices for pigs in the study area

Husbandry practice	Frequency (%)
Housing	
Always caged	17 (65.4)
Partially caged	7 (26.9)
Free–range	2 (7.7)
Disposal of pig faeces	
Used as manure	13 (54.2)
Washed into drains	9 (37.5)
Left in the cage	2 (8.3)
Disinfection protocols after leaving pigsty	
Yes	3 (11.5)
No	23 (88.5)
Use of protective boot when entering pigsty	
Yes	7 (27.0)
No	19 (73.0)
Use of glove when entering pigsty	
Yes	3 (11.5)
No	23 (88.5)
Use of face mask when entering pigsty	
Yes	0 (0.0)
No	26 (100)
Use of goggle when entering pigsty	
Yes	0 (0.0)
No	26 (100)
Availability of dedicated farm dress for entering pigsty	
Yes	8 (30.8)
No	18 (69.2)
Deworming of pigs	
Yes	0 (0.0)
No	26 (100)

### Farmers practices encouraging transmission of balantidiosis

Of the 24 pigsties, entry was restricted to only 12.5 %; most of the farmers infected with *B. coli* entered the pigsties twice a day (65.3 %) while others entered the pigsties once (19.2 %), thrice (11.5 %), and only one reported entering the pigsty an average of four times a day (3.8 %). Even though the farmers reported getting soiled with pig droppings in the pigsties, 19.2 % and 57.7 % of the farmers did not wash or wash their farming clothing only once in a week, respectively. It was also observed that 42 % of people that were not farmers came into contact with the farmers clothing as both spouses and children were involved in washing the farmers’ clothing. Interestingly, 88.5 % of either the farmers or their close contacts washed the farming apparel with their bare hands. Surprisingly, only one farmer indicated disinfecting clothes with domestic bleach prior to washing and only 11.5 % use protective gloves to wash the soiled clothing (Table 5).

**Table 5:** Farmers personal hygiene practices and diarrhea presentation

Practices	Frequency (%)
Restricted entry into pigsty	
Yes	3 (12.5)
No	21 (87.5)
Getting soiled with pig faeces when cleaning pigsty	
Yes	26 (100.0)
No	0 (0.0)
Average number of times, in a day, farmer enters pigsty	
1	5 (19.2)
2	17 (65.3)
3	3 (11.5)
4	1 (3.8)
Frequency of washing farm clothing, in a week	
None	5 (19.2)
Once	15 (57.7)
Twice	6 (23.1)
Person that washes farm clothing	
Self	15 (57.7)
Spouse	7 (26.7)
Child	4 (15.3)
Disinfecting of farm clothing with domestic bleach before washing	
Yes	1 (3.8)
No	25 (96.2)
Using protective glove to wash farm clothing	
Yes	3 (11.5)
No	23 (88.5)

### Balantidiosis infection according to haematological and biochemical parameters

Table 6 reports the various biomarkers analysed in this study. One-way analysis of variance (ANOVA) was used to test the differences in the mean levels of biomarkers analysed in both *B. coli* mono-infection and co-infection with other parasites, while *B. coli* negative individuals were used as the noninfected comparative group. In both mono- and co-infections, significant differences were observed in haemoglobin (p = 0.088), red blood cells (p = 0.033), mean cell volume (p = < 0.001), total white blood cells (p = 0.004), lymphocytes (p = 0.002), platelet count (p = 0.010), sodium ions (p = 0.02), chloride ions (p = 0.018), bicarbonate ions (p = 0.025) and albumin (p = 0.004) levels. Haemoglobin levels were found to be significantly reduced in both mono- and co-infections, co-infection levels being significantly lower than mono-infection cases. It was also observed that red blood cell levels were significantly lower among co-infected individuals than in mono-infected ones. However, mean cell volume (MCV) was significantly elevated in co-infection compared to mono-infection. Again, it was observed that total white blood cells were significantly higher in infected cases than the noninfected group. Post hoc analysis indicated significantly higher levels of total white blood cells in mono-infections than co-infections. Lymphocyte levels were significantly elevated in co-infection but not in mono-infections, while platelet levels were significantly reduced in co-infection. Furthermore, sodium and chloride ions were reduced in the infection state, but mono-infection levels did not differ significantly from co-infection levels. Bicarbonate ions were also reduced in the infection state with co-infection levels significantly lower than mono-infection. Albumin levels were also found to be significantly lower in the infection state, while co-infection levels did not differ from mono-infection levels.

**Table 6:** Biomarkers associated with *B. coli* mono-infection

Biomarkers	*B. coli* negative individuals (n = 86)	*B. coli* mono–infections (n = 34)	*B. coli* and other parasites co–infection (n = 9)	p–value
Haemoglobin (g/dL)	13.4 ± 1.09^†^	11.6 ± 2.33^†^	10.7 ± 2.98^†‡^	0.008
Red blood cells (x10^12^/L)	3.87 ± 1.01^†^	3.14 ± 0.87^†^	3.04 ± 1.21^†‡^	0.033
Mean cell volume (fL)	85.3 ± 7.23^†^	103.3 ± 5.78^†^	109.3 ± 3.99^†‡^	< 0.001
Total white blood cells (x10^9^/L)	9.5 ± 2.2^†^	12.4 ± 3.4^†‡^	11.5 ± 2.5^†‡^	0.004
Neutrophils (%)	45.7 ± 7.2	42.7 ± 5.4	43.6 ± 6.4	0.087
Lymphocytes (%)	44.6 ± 6.4	42.2 ± 4.7^‡^	53.2 ± 2.7^‡^	0.002
Mid–cells (%)	7.2 ± 2.9^‡^	8.6 ± 4.0^‡^	11.3 ± 3.3^‡^	0.009
Platelet count (x10^12^/L)	151 ± 28^‡^	145 ± 35^‡^	112 ± 15^‡^	0.010
Sodium (mmol/L)	138.9 ± 5.4^†^	132.4 ± 3.6^†^	131.4 ± 3.7^†^	0.020
Potassium (mmol/L)	5.9 ± 0.54	5.5 ± 0.78	5.3 ± 1.01	0.106
Chloride (mmol/L)	93.7 ± 5.1^†^	89.3 ± 9.6^†^	88.3 ± 5.9^†^	0.018
Bicarbonate (mmol/L)	23.3 ± 2.31^†^	19.56 ± 1.89^†^	17.4 ± 2.01^†‡^	0.025
Urea (mmol/L)	5.6 ± 2.3	6.1 ± 1.8	5.9 ± 2.1	0.197
Creatinine (µmol/L)	75.4 ± 6.7	71.3 ± 5.0	72.5 ± 3.6	0.213
ALP (U/L)	205 ± 18.6	211 ± 20.1	198 ± 14.9	0.088
Total protein (g/L)	73.2 ± 4.7	69.2 ± 3.5	67.6 ± 6.6	0.077
Albumin (g/L)	36.4 ± 5.1^†^	34.9 ± 3.2^†^	34.0 ± 3.9^†^	0.004
AST (U/L)	33.8 ± 3.7	34.4 ± 4.0	35.5 ± 2.1	0.115
ALT (U/L)	31.5 ± 0.89	28.5 ± 2.5	33.5 ± 1.4	0.066
Indirect bilirubin (µmol/L)	5.3 ± 0.88	4.7 ± 1.07	6.1 ± 1.15	0.321
Direct bilirubin (µmol/L)	8.4 ± 0.56	9.8 ± 0.78	10.7 ± 0.57	0.228

Values of biomarkers presented as mean ± standard deviation, ^†^significant difference between infection (either mono or co-infection) and noninfected comparative group, ^‡^significant difference between co-infection and noninfected comparative group. *ALP* alkaline phosphatase, *AST* aspartate aminotransferase, *ALT* alanine aminotransferase

## Discussion

Balantidiosis is a neglected tropical disease, prevalent in rural communities where pig rearing is common [3]. This study reports the findings from the human balantidiosis survey in Ghana and reports for the first time the burden of human balantidiosis in two pig rearing communities in Ghana as well as the identification of probable at-risk groups. More importantly, the clinical attributes of balantidiosis in humans have been documented. In 2008, a study carried out in the Eastern Region of Ghana reported 13.6 % prevalence of *B. coli* in commonly consumed vegetables [1]. Cases of human balantidiosis have been reported in some tropical and subtropical countries. Indeed, human balantidiosis prevalence of 28, 29, and 12 % were reported in Papua New Guinea, Bolivia and Venezuela, respectively [9, 12, 13]. In addition, in China [14], Peru [15], and India [16], cases of balantidiosis in human were described. However, in Ghana, a cross-sectional study, carried out in 2020, in the Ga West Municipality in the Greater Accra Region of Ghana identified asymptomatic *B. coli* infection in nine individuals in Opah and Onyansana communities [10]. Therefore, this study was purposively designed to unearth the real burden of infection in these two communities.

A high prevalence of human balantidiosis (10.4 %) was found in the Opah and Onyansana communities. The prevalence of *B*. *coli* infection was similar in the two study sites as well as among the age classes and between males and females. Similar rates of infection were also observed in participants with different educational levels, even though the risk of infection was insignificantly higher in participants with no formal education. However, infection status was significantly associated with pig farmers even though some exposed household members were infected. This observation confirms earlier hypotheses that pig rearing is a high-risk venture for *B. coli* transmission in humans [17]. Also, males and individuals without formal education were mostly infected, likely because more males than females are normally engaged in various farming activities and are mostly within the active working force [18–20]. Moreover, individuals without formal education were less informed about healthy lifestyles and lacked appreciation of control and prevention of parasitic diseases [21]. Although significant number of the farmers and their exposed household members were found to be infected by *B. coli* and other parasites, the wet preparation and formol ether concentration techniques could exhibit low sensitivity. Saline wet preparation is also inefficient in detecting low levels of parasites [22], while in the case of the concentration technique, the gauze used for filtration could trap the *B. coli* cysts resulting in false negative in cases of low cyst count.

In this study, poor and unhealthy farming practices were reported and could favour transmission of the parasite among farmers and their exposed household members. Indeed, the majority of the farmers neither washed their farm clothing regularly nor decontaminate their clothing prior to washing them. Moreover, almost 70 % of the farmers did not have dedicated farm clothing, thus increasing the risk for several clothes to be contaminated by pig faeces and subsequently contaminate other items in the household. Moreover, some of the pig farms operate either free-range or partially confined systems. This poor husbandry practice could contaminate the environment with infected pig faeces and subsequently facilitate the dissemination of *B. coli* parasites to individuals that are not directly involved in pig rearing in the community. In this study, these poor husbandry practices led to the infection of almost 6.0 % of the exposed household members with *B. coli*. Moreover, free-range pigs are more infectious that caged animals [23], thus representing a greater risk to the community when they are allowed to wander. The study also observed that there was no restricted entry into the majority of the pigsties and household members handled soiled farm clothing with bare hands without proper training or coaching on how to hygienically wash contaminated protective clothing. In a previous study, living close to pig farms and contamination of wells, streams, rivers, ponds, and soil with *B. coli* cysts have been reported as factors associated with the infection [23]. Moreover, *B. coli* cysts are resistant to sodium hypochlorite, a common bleach used to decontaminate clothing [24].

Regarding the clinical features of balantidiosis, the reduction in haemoglobin and red blood cells as observed in this study is widespread in most pathogenic entero-protozoa infections [25]. Even though bloody stool is common with balantidiosis [26], in this study only about 12 % of the *B. coli* infection presented with visible blood in stool samples. Additionally, occult blood was detected in all infected stool samples. Visible blood was not observed in almost all infected stool samples, possibly because the infected individuals did not exhibit fulminant infection, which culminates in extensive inflammation of the colon. Reduction in haemoglobin and red blood cell levels together with increased mean cell volume was suggestive of megaloblastic anaemia mostly caused by deficient of vitamin B-12 and /or folate. Similar findings were observed in *B. coli* infected patients in Venezuela [27] and recently in Thailand [28]. Significant thrombocytopenia was evident in *B. coli* infected individuals which was worsened in co-infection state. Furthermore, a reduction in levels of sodium, chloride and bicarbonate ions was observed in the infected individuals as was previously reported by Yu et al. [14]. In that case report, mild anaemia, hypokalemia, hyponatremia, and hypochloremia were observed as well as occult blood in stool samples. Reduction in these ions could be as a result of diarrhea experienced by all infected individuals, while the presence of occult blood in all studied individuals could be as a result of parasite-induced colitis [14]. Reduction in albumin levels confirms the association of malnutrition with balantidiosis, as highly documented in several parasitic studies [9, 29–31].

This study did not screen the infected individuals and noninfected comparative group individuals for haemo-parasites (e.g., malaria, babesia, filarial worms etc.), sickle cells, and other microbiological markers such as HIV. Furthermore, the oxidative stress and glucose-6-phosphate dehydrogenase (G6PD) statuses of the individuals were unknown. These could affect the levels of the biomarkers reported in this publication. Furthermore, clinical presentations such as anorexia, fever, nausea, and abdominal pain were not investigated in this study. These limitations should be considered in future studies.

## Conclusions

Human balantidiosis is endemic in two pig-rearing communities in Ga West Municipality, Ghana, with an overall prevalence of 10.4 %, with direct pig farmers being significantly infected. Most of the people infected with *B. coli* produced semi-formed, loose and blood stools. Irrespective of the physical appearance of their stool samples, occult blood was present. Individuals infected with *B. coli* exhibited relative reductions in haemoglobin, red blood cells, platelet count, lymphocytes, chloride, bicarbonate, and albumin, while mean cell volume levels significantly increased. Continuous surveillance for *B. coli* infection in endemic areas is essential for early treatment to prevent pathologies associated with infected individuals.

## Data Availability

The datasets generated and/or analysed during the current study are available in the Dataverse repository, 10.7910/DVN/EF9USF.

## Abbreviations

ALP: Alkaline phosphatase
ALT: Alanine aminotransferase
ANOVA: Analysis of variance
AST: Aspartate aminotransferase
CBSV: Community-based surveillance volunteer
FEC: Formol ether concentration
fL: Femtolitre
G6PD: Glucose-6-phosphate dehydrogenase (G6PD)
HIV: Human immunodeficiency virus

## References

[1] Kudah C, Sovoe S, Baiden F (2018). Parasitic contamination of commonly consumed vegetables in two markets in Ghana. Ghana Med J.

[2] Permin A, Yelifari L, Bloch P, Steenhard N, Hansen NP, Nansen P (1999). Parasites in cross-bred pigs in the Upper East Region of Ghana. Vet Parasitol.

[3] Schuster FL, Ramirez-Ávila L (2008). Current world status of Balantidium coli. Clin Microbiol Rev.

[4] Vásquez W, Vidal J (1999). Colitis balantidiásica: a propósito de un caso fatal en el departamento de Huancavelica. Anales Facul Med Sao Marcos.

[5] Parija SC. The ciliate protozoan, protozoan of undetermined taxonomic status. Textbook of medical parasitology. 4th ed. New Delhi: All India Publishers and Distributors; 2013.

[6] Kumar M, Rajkumari N, Mandal J, Parija SC (2016). A case report of an uncommon parasitic infection of human balantidiasis. Trop Parasitol.

[7] Centre for Disease Control and Prevention. Balantidiasis. 2013; https://doi.org/www.cdc.gov/parasites/balantidium.

[8] Aryee SND, Osei-Amponsah R, Adjei OD, Ahunu BK, Skinner BM, Sargent CA (2019). Production practices of local pig farmers in Ghana. Int J Livestock Prod.

[9] Esteban J-G, Aguirre C, Angles R, Ash LR, Mas-Coma S (1998). Balantidiasis in Aymara children from the northern Bolivian altiplano. Am J Trop Med Hyg.

[10] Aninagyei A, Yirenkyi R, Rufai T, Chandi MG (2020). Enteroparasitism in hard-to-reach community dwellers: a cross-sectional study in Ga West Municipality in Ghana. J Parasitol Res.

[11] Macfarlane SB (1997). Conducting a descriptive survey: 2. Choosing a sampling strategy. Trop Doct.

[12] Owen LL (2005). Parasitic zoonoses in Papua New Guinea. J Helminthol.

[13] Devera R, Requena I, Velasquez V, Castillo H, Guevara R, De Sousa M (1999). Balantidiasis in a rural community from Bolivar State. VenezuelaBol Chil Parasitol.

[14] Yu P, Rong J, Zhang Y, Du J (2020). Dysentery caused by Balantidium coli in China. Korean J Parasitol..

[15] Gomez Hinojosa P, Espinoza-Ríos J, Carlin Ronquillo A, Pinto Valdivia JL, Salas Dueñas Y, Zare Morales W (2019). Colonic balantidiasis: report of a fatal case and review of the literature. Rev Gastroenterol Peru.

[16] Sukhpreet K, Gupta A (2016). Urinary Balantidiasis: a rare incidental finding in a patient with chronic obstructive pulmonary disease. J Cytol.

[17] Damriyasa IM, Bauer C (2006). Prevalence and age-dependent occurrence of intestinal protozoan infections in suckling pigs. Berl Mu¨nchTiera¨ztl Wochenschr.

[18] Dada EO (2016). Prevalence of human intestinal helminthes among primary school children in Ipogun, Ifedore local government area Nigeria. J Glob Biosci.

[19] Abide M, Nibret E, Munshea A (2017). Prevalence of intestinal helminthic infections and malnutrition among school children of the Zegie Peninsula, northwestern Ethiopia. J Infect Public Health.

[20] Gebretsadik G (2016). Prevalence of intestinal parasites and associated risk factors among school children of Homesha District (Woreda) in Benishangul-Gumuz regional State, western Ethiopia. J Family Med Health Care.

[21] Dhanabal J, Selvadoss PP, Muthuswamy K (2014). Comparative study of the prevalence of intestinal parasites in low socioeconomic areas from South Chennai, India. J Parasitol Res.

[22] Mengist HM, Demeke G, Zewdie O, Belew A (2018). Diagnostic performance of direct wet mount microscopy in detecting intestinal helminths among pregnant women attending ante-natal care (ANC) in East Wollega, Oromia, Ethiopia. BMC Res Notes.

[23] Karanis P, Kourenti C, Smith H (2007). Waterborne transmission of protozoan parasites: a worldwide review of outbreaks and lessons learnt. J Water Health.

[24] Hampton J, Spencer PBS, Elliot AD, Thompson RCA (2006). Prevalence of zoonotic pathogens from feral pigs in major public drinking water catchments in Western Australia. EcoHealth.

[25] De Domenico I, McVey Ward D, Kaplan J (2008). Regulation of iron acquisition and storage: consequences for iron-linked disorders. Nat. Rev. Mol. Cell Biol.

[26] Yazar S, Altuntas F, Sahin I, Atambay M (2004). Dysentery caused by Balantidium coli in a patient with non-Hodgkin’s lymphoma from Turkey. World J Gastroenterol.

[27] Rosirisa CJ, Isabela HC, Orlandoa U, Javiera P, Mariob R, Norkac B (2003). Balantidium coli in an HIV-infected patient with chronic diarrhoea. AIDS.

[28] Martviset P, Sirisabhabhorn K, Pumpa S (2020). Urinary balantidiasis in a patient with systemic lupus erythematosus and lupus nephritis: a case report. J Med Case Reports.

[29] Prenner G, Wasler A, Fahrleinter-Pammer A (2014). The role of serum albumin in the prediction of malnutrition in patients at least five yr after heart transplantation. Clin Transplant.

[30] Abide M, Nibret E, Munshea A (2017). Prevalence of intestinal helminthic infections and malnutrition among school children of the Zegie peninsula, northwestern Ethiopia. J Infect Public Heal.

[31] Rajoo Y, Ambu S, Lim YAL, Rajoo K, Tey SC, Lu CW, Ngui R (2017). Neglected intestinal parasites, malnutrition and associated key factors: a population based cross-sectional study among indigenous communities in Sarawak, Malaysia. PLOS ONE.

